# Effects of Molybdenum Addition on Rolling Contact Fatigue of Locomotive Wheels under Rolling-Sliding Condition

**DOI:** 10.3390/ma13194282

**Published:** 2020-09-25

**Authors:** Yanpeng Wang, Pengcheng Xiang, Haohao Ding, Wenjian Wang, Qiang Zou, Xuehua Liu, Jun Guo, Qiyue Liu

**Affiliations:** 1 Tribology Research Institute, State Key Laboratory of Traction Power, Southwest Jiaotong University, Chengdu 610031, China; wendellsy@163.com (Y.W.); 17726463996@163.com (P.X.); wwj527@swjtu.cn (W.W.); guojun@swjtu.cn (J.G.); liuqy@swjtu.cn (Q.L.); 2 Technology Center, Ma’anshan Iron and steel Co., Ltd., Ma’anshan 243000, China; zouqiang0243@sohu.com (Q.Z.); 15755519332@163.com (X.L.)

**Keywords:** locomotive wheel material, molybdenum element, RCF life, RCF crack, shelling

## Abstract

Rolling contact fatigue (RCF) damages often occur, sometimes even leading to shelling on locomotive wheel treads. In this work, the RCF damage behaviors of two locomotive wheel materials with different molybdenum (Mo) contents were studied, and the influence of depth of wheel material was explored as well. The result indicates that with the increase in the Mo content from 0.01 wt.% (wheel 1, i.e., a standard wheel) to 0.04 wt.% (wheel 2, i.e., an improved wheel), the proeutectoid ferrite content and the interlamellar spacing of pearlite decreased, the depth and length of the RCF cracks increased and the average RCF live of locomotive wheel steel improved by 34.06%. With the increase in the depth of material, the proeutectoid ferrite content and the interlamellar spacing of pearlite increased, the depth of RCF cracks increased, the length of RCF cracks of wheel 1 increased and then decreased whereas that of wheel 2 decreased, the RCF live showed a decrease trend for wheel 1, while the RCF life increased and then decreased for wheel 2. The processes of shelling can be divided into three patterns: cracks propagating back to the surface, crack connection and fragments of surface materials.

## 1. Introduction

Rolling contact fatigue (RCF) has played an important role in determining the operational reliability of the wheel/rail system [1]. As is well known, residual stresses and strain hardening occurred in the material near the wheel/rail contact after cyclic loading, which raised the elastic limit for wheel materials. This phenomenon was called *elastic shakedown* [2,3]. When the stress in the contact exceeded the elastic shakedown limit of wheel materials, permanent plastic deformation occurred for each subsequent wheel revolution. Once the material ductility was not sufficient to satisfy the accumulated deformation, the RCF cracks eventually began and grew. This phenomenon was called *ratchetting* [2,3]. The *elastic shakedown* and *ratchetting* were closely related to the RCF of wheels [4,5,6]. Based on these phenomena, many research has been conducted to clarify the RCF mechanism of wheels and to predict the RCF life using experimental and numerical methods [7,8,9,10,11].

**Figure 1:**
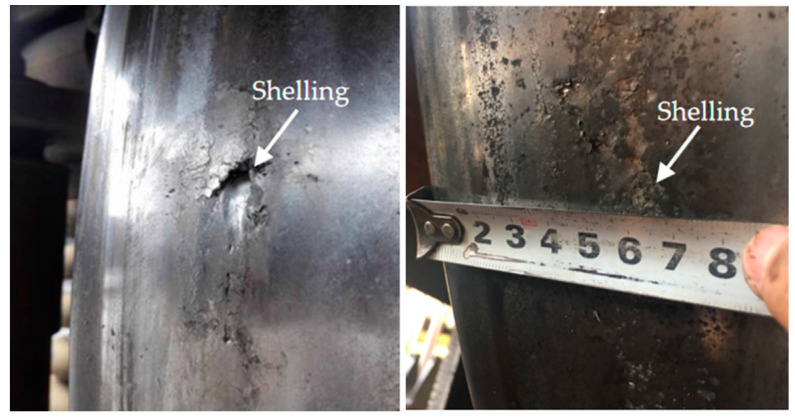
Shelling on locomotive wheel treads in China.

As one of the typical RCF failures, shelling often occurred on the locomotive wheel treads on the heavy haul railways in north China, as shown in Figure 1. This kind of RCF damage required frequent re-profiling, which shortened the wheel lives [2]. In general, shelling was the result of the continuing propagation of RCF cracks and happened after high-cycle fatigue [12,13]. Thus, the occurrence of shelling was considered to be the end of RCF life in some experimental studies [14,15]. Makino et al. [13] proposed that shelling was caused by branching cracks below 8 mm of surface, and that the RCF life decreased with the increase in slip ratio and contact stress. Kato et al. [14] reported that the RCF life decreased in some thermal cycle conditions because the elevated temperature (300 °C and 500 °C) decreased the material strength. Thus, it is important to find a way to alleviate shelling and improve the service life of wheels. At present, the mainly used methods in the field to alleviate shelling are early detection and timely re-profiling of wheels [16,17].

A change of alloying elements could alter the mechanical properties of wheel materials significantly. Various improved wheel materials with a good combination of strength and toughness have been developed [18,19,20]. Zeng et al. [18] have proved that the railway wheel steel with high contents of Si and Mn and low content of Cr had a good combination of strength and toughness. The improved wheel also showed better resistance to spalling caused by thermal-mechanical effects [19]. Zhang et al. [20] found that a nanostructured railway wheel made of Si-Mn-Mo-V low-carbon steel had better anti-fatigue performance. Molybdenum (Mo) has been widely used in many metallic materials, such as grey cast iron [21], powder metallurgy [22] and high-speed steel [23]. The traditional wheel materials have a ferrite-pearlite (proeutectoid ferrite and pearlite) microstructure. The addition of Mo in the wheel resulted in high hardness due to the finer pearlite lamellae spacing and the high toughness due to the finer grains [2,3]. But the high proportion of Mo could also increase the risk of temper brittleness [2]. The present study focused on the effect of a small amount of Mo on the RCF life of locomotive wheel steel.

In the industrial field, tensile and impact tests were the general methods of evaluating the mechanical properties of railway wheel materials, during which the microstructures were observed, and the stress-strain curves and impact absorbed energy were analyzed [18,20,24]. However, the key to the wheel RCF was the accumulation of plastic deformation under the cyclic rolling-sliding condition. Therefore, it is necessary to evaluate the RCF property of wheel materials using the rolling-sliding tests as a supplement. In the experiments, the RCF damage could be accelerated by adding liquid into the interface of wheel/rail contact [14,15,16]. Furthermore, previous studies proposed that shelling was the result of deteriorating RCF cracks [12,13,14,15,16], but the generation processes of shelling have not been explored systemically.

In the present study, the RCF processes of two locomotive wheels with different Mo contents were explored via the twin-disc tests. The influences of Mo content and the depth of material on the RCF lives were studied. Besides, the processes of shelling were discussed in detail.

## 2. Experimental Details

### 2.1. Experimental Specimen

In this study, two kinds of locomotive wheels (wheel 1, a type of standard wheel and wheel 2, a type of improved wheel) with different alloying elements were examined. The two wheels were produced through the following processes: smelting of steel, casting, stamping, rolling, dimension and heat treatment. The heat treatment process included: heating the wheels to 830~850 °C for 1.5 h, followed by water quenching to 350 °C, then tempering at 520 °C for 1 h and then cooling to room temperature. The steel grade of wheel 1 was J1, which was a kind of locomotive wheel material according to the Chinese railway industry standard TB/T 3469-2016. The main difference was that wheel 1 contained 0.01 wt.% Mo and wheel 2 contained 0.04 wt.% Mo. In the production process, composition analysis was carried out two times during the smelting progress. Meanwhile, composition analysis was also conducted for wheels after production. Therefore, the error of Mo content in wheels was controlled within 0.01 wt.%. The chemical compositions of the wheel materials are listed in Table 1. The mechanical properties of the two wheels measured according to the standard TB/T 3469-2016 are shown in Table 2. The yield strength (R_eH_) referred to the upper yield point and the ultimate tensile strength (R_m_) referred to the highest point in the stress-strain curve. The yield strength and the ultimate tensile strength of wheel 2 were larger than those of wheel 1, but the impact toughness of the two wheels was similar. The matrix hardness of the two wheels was measured at 25 mm below the tread. The matrix hardness of wheel 1 was larger than that of wheel 2. 

**Figure 2:**
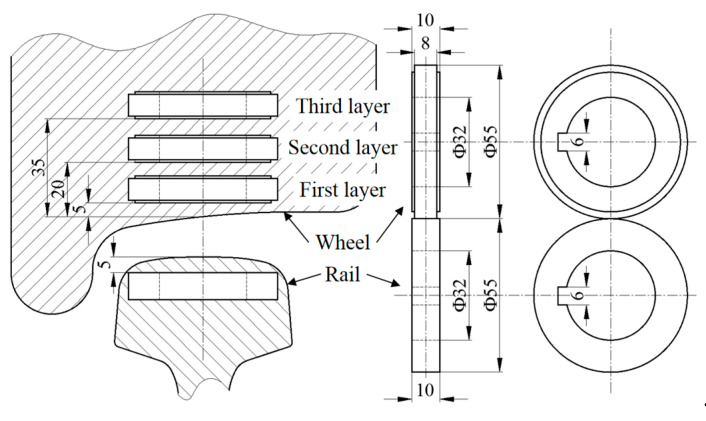
Sampling positions and sizes of the wheel and rail rollers (unit: mm).

The wheel rollers were cut from the two types of wheels and the rail roller was from a U75V railhead, with a diameter of 55 mm and a contact width of 8 mm. The contact surfaces were ground to reach a roughness (R_y5_) of about 2 μm. On each wheel, three rollers were cut from different depths (1st layer, 2nd layer and 3rd layer). The sampling position and sizes of the wheel and rail rollers are shown in Figure 2.

### 2.2. Experimental Apparatus and Parameters

A twin-discs test machine (MJP-30A rolling-sliding test rig) was used to conduct the RCF experiments in this study. Details of the test rig have been described in the previous work [25]. Two discs (i.e., the wheel roller and the rail roller) were driven by independent servo motors. Normal force was continually applied by a hydraulic loading system (measurement error: ±2%). The creep ratio of wheel and rail was achieved by alteration of the rotational speeds of servo motors. The creep ratio was positive to simulate a decelerating (braking) wheelset, i.e., the speed of the rail roller was slightly larger than the speed of the wheel roller. Vibration in the vertical direction was measured by the sensor fixed on the shaft of upper disc.

In all experiments, the rotational speed of the rail roller was 1500 rmp and the creep ratio was 0.3%. According to the Hertzian simulation rule [26], the normal force was set as 4600 N to produce the maximum contact pressure of about 1230 MPa. Water was supplied into the wheel/rail interface with a flow rate of 8 mL/s. Each test was finished when the amplitude value of the vibration was larger than 25 dB. The severe vibration was mainly caused by the shelling on the wheel roller, thus the cycle number when the vibration was larger than 25 dB was considered to be the RCF life of wheel roller. Each test was repeated three times. The experimental parameters are shown in Table 3.

The surfaces of wheel and rail rollers were observed via optical microscope (OM) (KEYENCE VHX-6000, Osaka, Japan). Then, longitudinal sections of the wheel rollers were cut, mounted in resin, ground to 2000 grit silicon carbide paper, polished and etched with natal (4% HNO_3_ in ethanol). The hardness was measured via the Victorinox hardness tester (MVK-H21, Akashi, Japan). The subsurface morphologies were inspected via optical microscope (OM) (KEYENCE VHX-6000, Osaka, Japan) and scanning electron microscope (SEM) (Phenom Pre-SE, The Netherlands). 

## 3. Results

### 3.1. Microstructures of Wheel Materials before Tests

**Figure 3:**
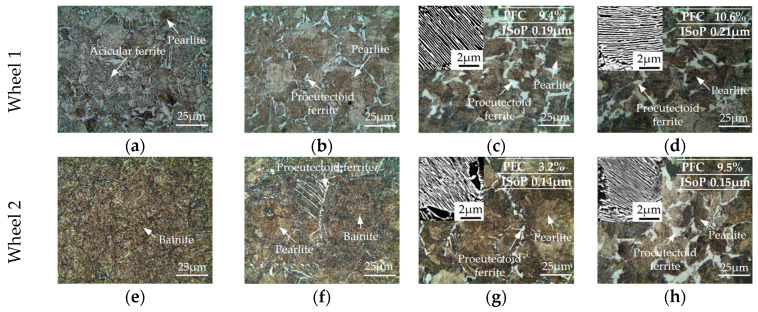
Microstructures of wheels: wheel 1 (standard wheel) at depths of (**a**) 2 mm, (**b**) 8 mm, (**c**) 25 mm and (**d**) 35 mm; wheel 2 (improved wheel) at depths of (**e**) 2 mm, (**f**) 8 mm, (**g**) 25 mm and (**h**) 35 mm.

There was only 0.03 wt.% difference in Mo contents between the two types of wheel materials. However, the slight difference in Mo content could lead to obvious differences in the microstructures, especially the microstructure near the tread surface, as shown in Figure 3. In addition, the microstructure changed with the sampling depth on each wheel, because the cooling rate decreased with the depth in the wheel rim when air chilling during the production. Therefore, there were usually “complex layers” on the tread surface [18] which were different from the matrix because the critical cooling rate for pearlite formation was exceeded at that position [26].

It is seen from Figure 3a,e that at 2 mm depth, the microstructure of wheel 1 (standard wheel) consisted of a small amount of pearlite and a large amount of acicular ferrite, whereas the microstructure of wheel 2 (improved wheel) mainly consisted of bainite. This is because the presence of Mo decreased the transformation temperature of bainite [27], thus a bainite layer was formed on the near-surface of wheel 2. At a depth of 8 mm (1st layer for wheel roller, Figure 3b,f), the combination of bainite and ferrite-pearlite was observed in wheel 2, whereas only ferrite-pearlite existed in wheel 1. As Figure 3c,d,g,h illustrate, at the depth of 25 mm (2nd layer) and 35 mm (3rd layer), the proeutectoid ferrite and pearlite structures were observed, which is the typical matrix structure of wheel materials. For the matrix structure, the proeutectoid ferrite content (PFC) and the interlamellar spacing of pearlite (ISoP) of wheel 2 were smaller than those of wheel 1. With the increase in depth, the proeutectoid ferrite content (PFC) and the interlamellar spacing of pearlite (ISoP) increased in two-wheel materials. 

In addition, the top layers near the surface (i.e., the “complex layers” on the tread surface) were too thin, and the wheels were re-profiled before application to remove the top layers. Thus the “complex layers” were not further explored via rolling-sliding tests. The wheel rollers for rolling-sliding tests were cut from the 1st, 2nd and 3rd layers.

### 3.2. Hardening of Wheel Materials after Test 

**Figure 4:**
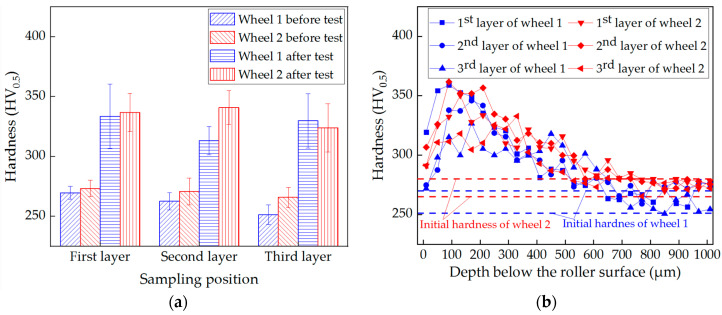
Hardness of wheel 1 (standard wheel) and wheel 2 (improved wheel): (**a**) surface hardness before and after tests; (**b**) hardness on cross section after tests.

The surface hardness of wheel rollers before and after tests is shown in Figure 4a. Before tests, the hardness of wheel 2 (improved wheel) was higher than that of wheel 1 (standard wheel). The higher hardness of wheel 2 was due to the decrease in the proeutectoid ferrite content and the pearlite interlamellar spacing compared with those of wheel 1 [3,28]. Furthermore, before tests, the hardness decreased slightly with the increase in depth, which is a common phenomenon for integrated wheel steel because of the inconsistent cooling rate during heat treatment [18]. After the test, the surface hardness of wheel rollers was improved to the range of 313~341 HV_0.5_. 

In order to study the hardening phenomenon, the hardness was measured on the cross sections after tests, as shown in Figure 4b. With the increase in depth, the hardness decreased. Because the deformation layer usually occurred on the surface under the rolling-sliding condition. Besides, the hardness remained relatively stable at a depth of around 600–700 μm. The influences of the Mo content and the sampling depth on the hardness after tests are not obvious. 

### 3.3. RCF Lives of Wheel Materials 

**Figure 5:**
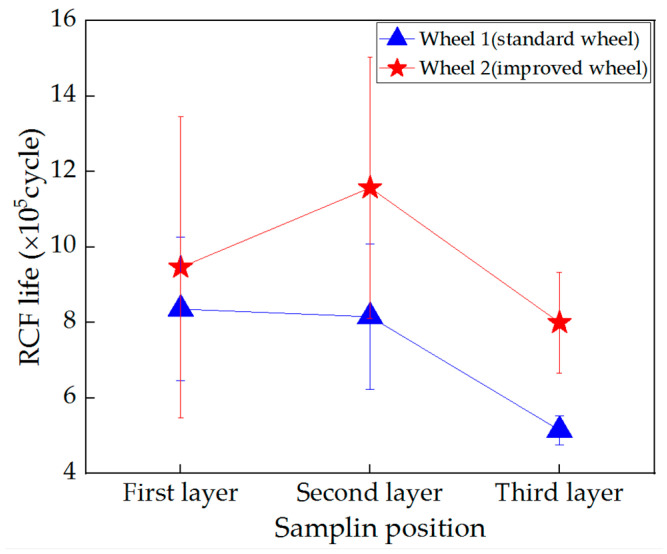
Rolling contact fatigue lives of the wheel rollers.

Figure 5 shows the RCF life of each wheel roller. The RCF lives of wheel 2 (improved wheel) were larger than those of wheel 1 (standard wheel). This indicates that wheel 2 had better RCF resistance than wheel 1. Comparing the RCF lives of wheel rollers at different sampling depths, the second layer of wheel 2 had the longest RCF life, while the RCF life of wheel 1 had a decreasing trend with the depth. Moreover, it is obvious that the RCF lives of the third layer rollers were the smallest.

Specifically, the RCF lives of wheel 1 were around 836,000, 815,000 and 514,000 cycles at different depths, and the RCF lives of wheel 2 were around 946,000, 1,157,000 and 800,000 cycles. Thus, the addition of Mo could improve the RCF life of the wheel; the increase rates were 13.23%, 41.90% and 55.46% at different depths, respectively. When RCF damage such as shelling occurs on locomotive wheels, the wheels need to be re-profiled, leading to a reduction in wheel diameter. When the diameter of the wheel is reduced to 1150 mm, the wheel will be replaced. In order to compare the RCF lives of two wheels in the whole service process, the average RCF lives of the wheel rollers with different depths were calculated. The average RCF life of wheel 1 (standard wheel) was 722,000 cycles, and the average RCF life of wheel 2 (improved wheel) was around 968,000 cycles. The increase rate was 34.06%.

### 3.4. Damages of Wheel Materials

#### 3.4.1. Surface Morphology

**Figure 6:**
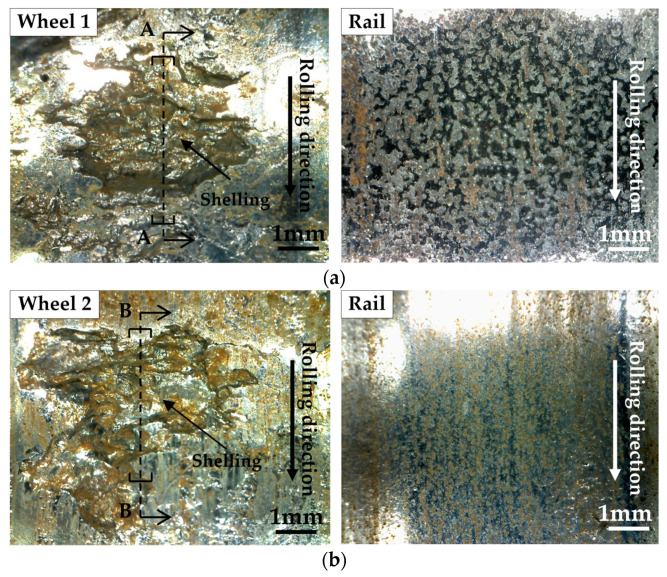
Surface damage on wheel rollers (second layer) and rail rollers after tests: (**a**) wheel 1 (standard wheel) test; (**b**) wheel 2 (improved wheel) test.

After the test, shelling occurred at one or more places on the wheel roller surface. The area of shelling ranged from 2 to 12 mm^2^ without significant regularity. There was no significant difference in the damage forms of rollers taken from different depths of the same wheel. The key issue of this study was to clarify the effect of Mo addition on RCF. Therefore, damage analysis was carried out on the second layer tests (with the longest RCF lives for both the wheel materials), as shown in Figure 6. The two kinds of wheels had little difference between them in terms of surface damage. Therefore, it was necessary to observe the damage on the cross sections. In addition, only slight fatigue cracks occurred on the rail rollers surface. This means that the severe vibration of 25 dB which was used to define the RCF life was caused by shelling damage on the wheel.

#### 3.4.2. Subsurface Morphology

**Figure 7:**
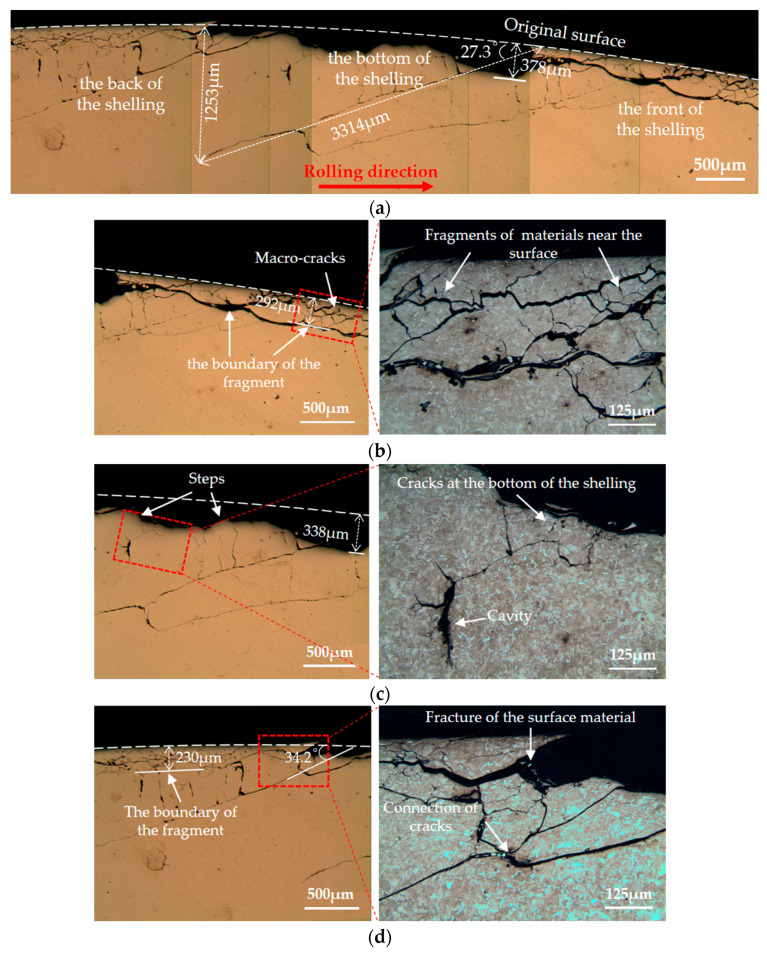
Subsurface damage around the shelling on wheel 1 (standard wheel): (**a**) the overall view; (**b**) the front, (**c**) the bottom and (**d**) the back of the shelling.

**Figure 8:**
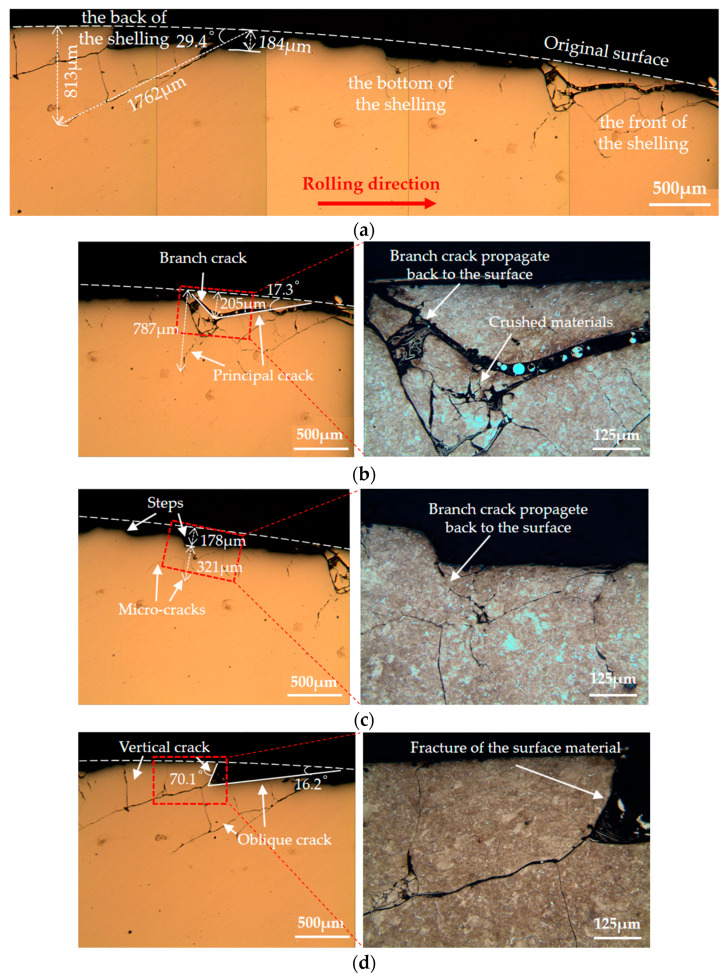
Subsurface damage around the shelling on wheel 2 (improved wheel): (**a**) the overall view; (**b**) the front, (**c**) the bottom and (**d**) the back of the shelling.

**Figure 9:**
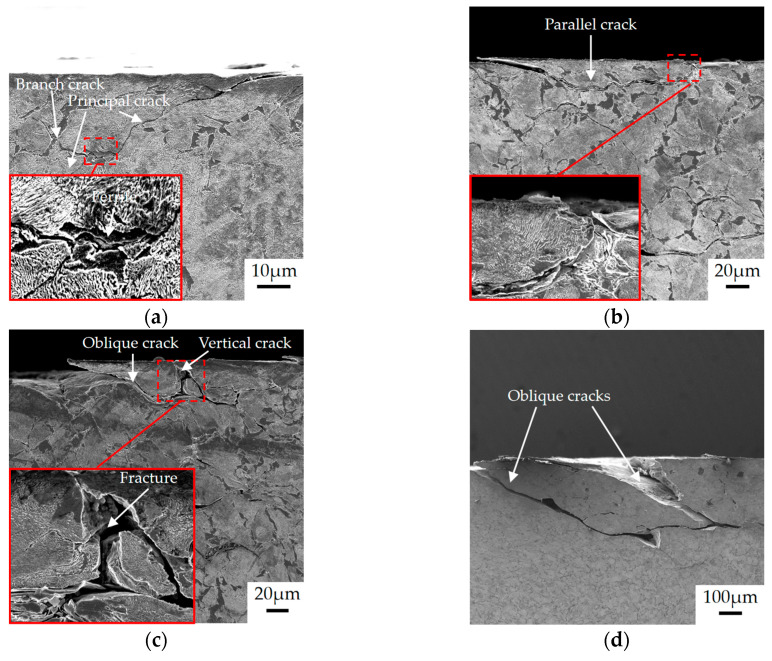
SEM images of cracks outside shelling: (**a**) branch crack grows back to surface; (**b**) parallel crack grows back to surface; (**c**) oblique and vertical cracks connect; (**d**) two oblique cracks connect.

The wheel rollers were cut along the rolling direction. The cutting line passed through the bottom of the shelling. Then the cross sections were observed, as shown in Figure 7, Figure 8 and Figure 9. Shelling and a large number of RCF cracks were observed. Along the rolling direction, the shelling area could be divided into three parts: the front, the bottom and the back of the shelling. Meanwhile, cracks with different angles were observed, thus they could be divided into three types in the present study: parallel cracks (0~10°), oblique cracks (10~70°) and vertical cracks (70~90°).

Figure 7a shows the overall view of the shelling on wheel 1, i.e., standard wheel (sectioned along the A-A line in Figure 6a). The maximum depth of shelling was around 378 μm. The crack with the largest size possessed a depth of around 1253 μm and a length of around 3114 μm, with an angle of 27.3° between the crack and original surface (oblique crack). Details of the damages at the front, the bottom and the back of shelling are shown in Figure 7b–d.

Figure 7b shows that at the front area of shelling, a large number of micro-cracks interlaced with each other, resulting in fragments of the materials near the surface. Below the fragments, as the boundary between fragments and the matrix, a parallel crack was formed at a depth of around 292μm. With an increase in the number of cycles, the fragments could separate the materials near the contact surface from the matrix, eventually increasing the area of the shelling. 

Figure 7c shows that the outline of the bottom of the shelling was not flat; there were several steps at the bottom. In addition to the shelling with a depth of 383μm, there were also cracks connected with each other below the bottom of the shelling. The materials between these cracks could break away from the matrix, resulting in an increase in the depth of shelling. The cavity occurred at the tip of the crack because of the high pressure of the water trapped in cracks.

Figure 7d shows that apart from large size cracks, the fragments of the material near the surface (e.g., within 230 μm below the surface) could also occur at the back of the shelling. The connection of multiple cracks could lead to the fracture of the material between cracks.

Figure 8a shows the overall view of the shelling on wheel 2, i.e., improved wheel (sectioned along the B-B line in Figure 6b). The maximum depth of shelling was around 184 μm. The crack with the largest size possessed a depth of around 813 μm and a length of around 1762 μm. Details of the damages at the front, the bottom and the back of the shelling are shown in Figure 8b–d. 

Figure 8b shows that at the front area of the shelling, the surface crack branched after extending downward to around 205 μm from the surface. Then the principal crack continued to propagate down to 787 μm, while the branch crack propagated back to the surface. The angle between the crack and the original surface was 17.3°. The branch crack that propagated to the surface could cause material above the crack to break away from the matrix. The materials under the crack could be crushed by extrusion in the process of cyclic rolling. This damage could reduce the stability of the surface material.

Figure 8c shows that there were also several steps at the bottom of shelling. Tiny cracks occurred at the bottom of the shelling at a depth of around 321 μm. Branch cracks propagated back to the bottom of shelling. The material above these branch cracks could break away from the matrix, leading to an increase in the depth of shelling.

As shown in Figure 8d, there were a large number of oblique cracks and vertical cracks with large-size cracks at the back of shelling. From the outline at the back of the shelling, it can be inferred that the connection of the vertical crack (70.1°) and the oblique crack (16.2°) could lead to the fracture of materials above cracks. Undoubtedly, the continuous occurrence of fracture damage could also increase the size of the shelling.

By comparing the subsurface damage around the shelling of two kinds of wheels, we found that the main differences between wheel 1 (standard wheel) and wheel 2 (improved wheel) were: (i) the size and quantity of cracks in wheel 2 were smaller than those in wheel 1; (ii) there was milder fragment damage of materials near the surface in wheel 2 than in wheel 1.

There were also a large number of cracks at the surface far away from the shelling, as shown in Figure 9. As Figure 9a shows, the branch crack generated from an oblique crack when the principal crack propagated to proeutectoid ferrite. The branch cracks propagating to the surface were considered to be the cause of shelling [14]. Meanwhile, the parallel crack near the surface could also propagate to the surface, resulting in the removal of materials above the cracks, as shown in Figure 9b. Besides, when different cracks connected, the material between cracks could be separated from the surface, as shown in Figure 9c,d. In previous studies, the connection between the vertical crack and the oblique crack was also called “fracture” [29]. In summary, RCF cracks propagated in the above ways, which could lead to shelling.

#### 3.4.3. Statistics of RCF cracks

**Figure 10:**
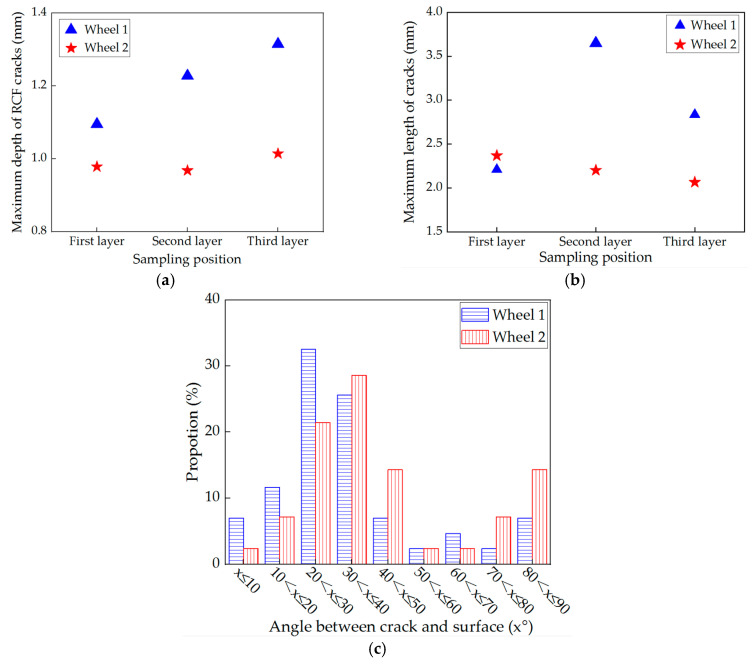
Statistics of RCF cracks on wheel 1 (standard wheel) and wheel 2 (improved wheel): (**a**) maximum depth and (**b**) maximum length of cracks; (**c**) distribution of crack angles.

The depth, length and angle of cracks on the wheel rollers were measured. Figure 10a shows that the maximum depth of RCF cracks is in a range of 0.8–1.4 mm. Meanwhile, the depth of RCF cracks of wheel 2 (improved wheel) is smaller than that of wheel 1 (standard wheel). The crack depth increased obviously on wheel 1, but increased slightly on wheel 2. 

The maximum lengths of the RCF cracks of the two wheels are similar at the first layer. At the second and third layers, the crack length of wheel 1 is longer than that of wheel 2, as shown in Figure 10b. With the increase in depth, the crack length first increased and then decreased for wheel 1, whereas it decreased slightly for wheel 2.

The distribution of RCF cracks angles is shown in Figure 10c. The crack angles on two wheels were mainly distributed in the range of 20~40°. The main difference was that the proportion of cracks with angles between 70~90° on wheel 2 was larger than that on wheel 1, while the proportion of cracks with angles of 0~10° on wheel 2 was smaller than that on wheel 1. This means that on wheel 2 (improved wheel), more fracture damage caused by vertical cracks occurred; while on wheel 1 (standard wheel), more fragment damage occurred with a boundary of long parallel cracks (Figure 8b,d).

## 4. Discussion

### 4.1. RCF of Wheel under Water Condition

**Figure 11:**
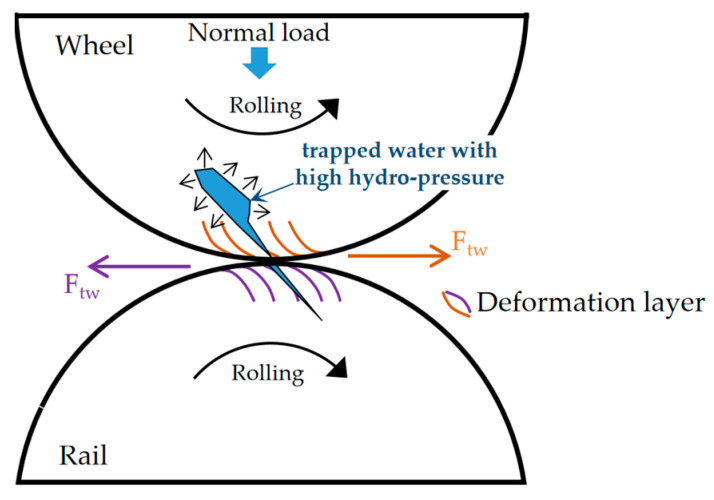
Schematic diagram of RCF crack propagation on wheel and rail rollers under the water condition adapted from [1].

The objective of this work was to investigate the RCF behaviors of two kinds of wheel materials, thus the water was added into the wheel/rail interface during the rolling-sliding tests to enhance the RCF damages. As shown in Figure 11, the directions of the plastic deformation layer and crack opening on wheel and rail rollers were determined by the tangential force of the wheel and rail discs (F_tw_ and F_tr_). In this study, the direction of the cracks on the wheel roller was consistent with the rolling direction, while the direction of the cracks on the rail roller was opposite. When cracks entered the wheel/rail contact zone, the openings of cracks were closed. The liquid in the crack on the wheel roller was sealed and trapped, but the liquid in the cracks on the rail roller was squeezed out. Then, under the cyclic normal load, the trapped liquid on the wheel roller accelerated the crack propagation due to high hydro-pressure [1,11]. Therefore, the RCF damage of the wheel roller was significantly more severe than that of the rail roller in this study (Figure 6).

### 4.2. The Process of the Shelling on Wheel

**Figure 12:**
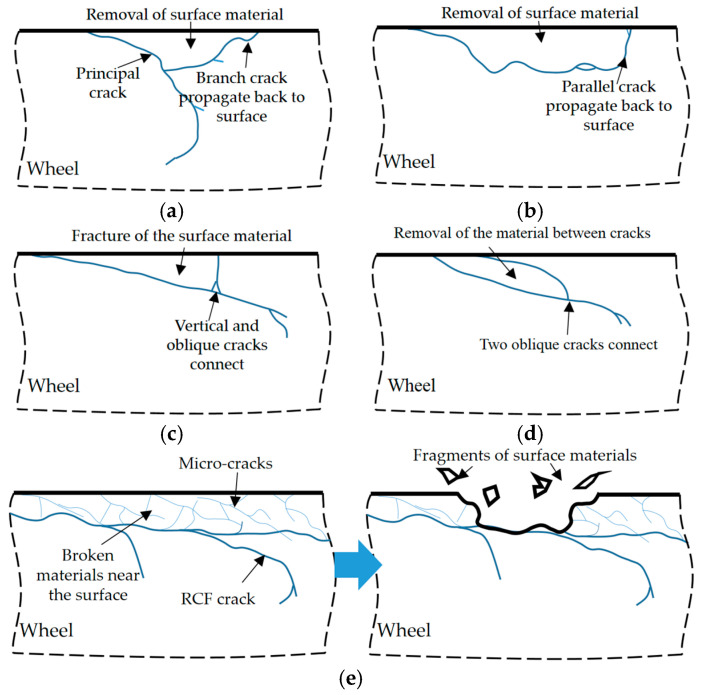
Schematic diagram of the processes of RCF damage: (**a**,**b**) cracks propagating back to surface, (**c**,**d**) crack connection, (**e**) fragments of surface materials.

In previous studies, the shelling processes were described as the propagation and union of adjacent cracks, or a result of branched cracks which propagated back to the surface [16,30]. But more shelling details were observed in the present study. According to the different forms of RCF cracks, the processes of shelling could be divided into three patterns, as shown in Figure 12: (i) cracks propagating back to the surface, (ii) crack connection and (iii) fragments of surface materials.

Concerning pattern (i): when branch cracks or parallel cracks propagated back to the surface, the material above cracks was removed from the matrix (Figure 12a,b), leading to the shelling of a piece of material. If the parallel crack was shallow, it was also called peeling [31]. Concerning pattern (ii): when two cracks connected—for example, a vertical crack and an oblique crack or two oblique cracks—the material between two cracks could be separated from the matrix (Figure 12c,d). Concerning the pattern (iii): when many tiny grid-like micro-cracks were generated near the surface as shown in Figure 7b, the material was divided into many small pieces. With the cyclic loading, these small fragments could be separated from the surface, leading to severe shelling (Figure 12e).

### 4.3. Influence of Mo Content on RCF

It was found in this study that RCF life was greatly improved by adding only a small amount of Mo into the wheel material. With the increase in Mo content from 0.01 to 0.04 wt.%, the RCF lives of the wheel rollers were increased by 13.23%, 41.90% and 55.46% at different depths of the wheel, and the average RCF life was increased by 34.06%. RCF life was mainly related to initiation and propagation of RCF cracks. With the increase in Mo content, the yield strength of the wheel was increased to 578 MPa (wheel 2, i.e., improved wheel). Therefore, the initiation of cracks was delayed. Moreover, the volume fraction of proeutectoid ferrite in wheel 2 (improved wheel) was smaller than in wheel 1 (standard wheel). The reduction of proeutectoid ferrite content was also helpful to alleviate the RCF cracks because the crack mainly initiated and developed along the ferrite [29,31]. Besides, Mo could delay the transition from austenite to pearlite during production, resulting in smaller pearlite lamellar spacing [27,28] of wheel 2 (Figure 3). The decrease in pearlite lamellar spacing could lead to a decrease in crack growth rate [32]. This was also the reason for the longer RCF life of wheel 2 compared to wheel 1. 

The RCF performance of wheel rollers at different sampling depths was also different. On the whole, the RCF life of wheel 1 decreased with the increase in sampling depth (Figure 5). This happened because with the increase in depth, the cooling rate was decreased and then the proeutectoid ferrite content was increased, which reduced the RCF life. For wheel 2, with the increase in sampling depth from the second layer to the third layer, the RCF life was decreased, but the RCF life of the first layer roller was also lower than that of the second layer roller. This was because the microstructure at a depth of 8 mm (Figure 3b, first layer) was composed of bainite and ferrite-pearlite, which might easily lead to the initiation of RCF cracks because of the inhomogeneous localized deformation behaviors [33]. 

It was also found that the form of RCF damage changed due to the increase in Mo content. As can be seen from Figure 8 and Figure 9, when shelling occurred, the sizes of cracks were larger in wheel 1 than wheel 2. As shown in Figure 3, at the same depth, the content of proeutectoid ferrite of wheel 2 was lower than that of wheel 1. Cracks were more likely to propagate along the ferrite with lower strength. Therefore, reducing the size and content of proeutectoid ferrite could increase the resistance of RCF cracks, eventually leading to fewer cracks and smaller crack sizes in wheel 2. 

In summary, improving the Mo content from 0.01% to 0.04% could increase the anti-fatigue properties of the wheel material, and the average RCF life was improved by 34.06%. The following study will focus on the RCF properties of wheel materials with a series of Mo contents to find the optimal wheel material.

## 5. Conclusions

In this work, the RCF damage behaviors and lives of two locomotive wheel materials with different Mo contents were studied via the rolling-sliding tests. Meanwhile, the influence of the depth of the wheel was also explored. The following conclusions can be drawn:

1. With the increase in Mo content from 0.01 wt.% (wheel 1, i.e., standard wheel) to 0.04 wt.% (wheel 2, i.e., improved wheel), the proeutectoid ferrite content and the pearlite interlamellar spacing were reduced. With the increase in depth, the proeutectoid ferrite content and the pearlite interlamellar spacing increased.
2. With the increase in Mo content from 0.01 to 0.04 wt.%, the RCF lives improved by 13.23%, 41.90% and 55.46%, at the first, second and third layers. With the increase in depth, the RCF lives increased and then decreased for wheel 2 (improved wheel), while the RCF lives showed a decreasing trend for wheel 1 (standard wheel).
3. Shelling and RCF cracks were generated on the wheel materials after the tests. The crack depth and length of wheel 2 (improved wheel) were smaller than those of wheel 1 (standard wheel). With the increase in the depth of the sampling position, the crack depth increased, whereas the crack length of wheel 1 first increased and then decreased and the crack length of wheel 2 decreased.
4. The processes of shelling could be divided into three patterns: cracks propagating back to the surface, crack connection and fragments of surface materials.

## Figures and Tables

**Table 1:** Chemical compositions of wheel and rail materials (wt.%).

Specimen	C	Si	Mn	P	S	Mo
Wheel 1 (Standard Wheel)	0.45~0.52	0.20~0.40	0.60~0.80	≤0.02	≤0.015	0.01
Wheel 2 (Improved Wheel)	0.45~0.52	0.20~0.40	0.60~0.80	≤0.02	≤0.015	0.04
Rail	0.71~0.80	0.50~0.80	0.70~0.15	≤0.03	≤0.03	-

**Table 2:** Mechanical properties of two wheels.

Specimen	Yield Strength (MPa)	Ultimate Tensile Strength (MPa)	Impact Toughness (J/cm^2^)	The MATRIX Hardness (HV_0.5_)
Wheel 1 (Standard Wheel)	533	879	28	257.6
Wheel 2 (Improved Wheel)	578	913	27	270.1

**Table 3:** Experimental parameters for all tests.

No.	Materials	Sampling Position	Speed (rmp)	Creep Ratio (%)	Normal Force (N)
1	Wheel 1 (Standard Wheel)	1st layer	1500	0.3	4600
2		2nd layer			
3		3rd layer			
4	Wheel 2 (Improved Wheel)	1st layer			
5		2nd layer			
6		3rd layer			
